# Pediatric tuina for allergic rhinitis in children: A systematic review and meta-analysis of randomized controlled trials

**DOI:** 10.3389/fped.2022.1043322

**Published:** 2022-11-14

**Authors:** Shifang Fu, Yuetong Li, Rongrong Li, Fengjiao Ren, Yinjing Piao, Yanguo Wang, Mingchi Luo

**Affiliations:** ^1^ Department of Massage and Rehabilitation, Second Teaching Hospital of Tianjin University of Traditional Chinese Medicine, Tianjin, China; ^2^ Department of Graduate School, Tianjin University of Traditional Chinese Medicine, Tianjin, China

**Keywords:** pediatric tuina, allergic rhinitis, children, randomized controlled trials, meta-analysis

## Abstract

**Aim:**

To evaluate the clinical efficacy of pediatric tuina for allergic rhinitis treatment in children.

**Methods:**

Three English, four Chinese, one Japanese, and two Korean databases were searched for relevant literature published till February 2021, and eligible randomized controlled trials (RCTs) were included for analysis. Data were screened and extracted independently using predesigned forms. The methodological quality evaluation was performed using the tool of Cochrane bias risk assessment, and meta-analysis was performed through Review Manager 5.3.

**Results:**

A total of 12 RCTs, which included 716 children, were selected for meta-analysis. Compared with Chinese herbal medicine, acupuncture, Western medicine, and other therapies, pediatric tuina alone or in combination with other treatments had a higher clinical effectiveness (relative risk = 1.16, 95% confidence interval [CI] = 1.08–1.25) in treating children with allergic rhinitis. Pediatric tuina also effectively improved the following signs and symptoms of allergic rhinitis in children: nasal congestion (mean difference [MD] = −0.44, 95% CI = −0.70 to −0.19), runny nose (MD = −0.39, 95% CI = −0.55 to −0.23), sneezing (MD = −0.23, 95% CI = −0.38 to −0.08), and turbinate swelling (MD = −0.26, 95% CI = −0.48 to −0.04); all differences were statistically significant.

**Conclusions:**

The present study provided favorable evidence for the treatment of allergic rhinitis in children with pediatric tuina. However, owing to the impact of research quality, this evidence needs to be validated *via* strictly designed clinical trials.

## Introduction

Allergic rhinitis (AR) is a noninfectious inflammatory disease mediated by immunoglobulin (Ig) E and the most common chronic disease in children (1). Children with AR exhibit several symptoms, including sneezing, watery rhinorrhea, nasal congestion, and itching (2). The International Research Organization for Childhood Asthma and Allergy systematically assessed the prevalence of allergic diseases in 98 countries, and the results revealed that the overall prevalence of AR in children aged 6–7 and 13–14 years was 8.6% and 14.6%, respectively (3, 4). AR not only has a negative impact on the physical and mental health, quality of life, and learning ability of children but may also lead to potential complications such as sinusitis, otitis media, and asthma (5, 6). In addition, children with AR may place a heavy burden on the family and society (7). Western medicines for the treatment of children with AR primarily comprise oral antihistamines, intranasal corticosteroids, decongestants, and leukotriene receptor antagonists (8–10). Owing to their poor compliance and obvious side effects (11, 12), some parents attempt to find other alternative therapies to relieve the symptoms of children with AR. Nondrug therapies, particularly pediatric tuina, have become a feasible strategy for the treatment of children with AR because of the advantages of safety, low cost, and easy acceptance by children (13, 14).

Pediatric tuina is an external treatment method guided by the basic principles of traditional Chinese medicine. According to the physiological and pathological characteristics of children, various techniques, such as pushing, pinching, and pressing, are used on the specific parts of a child's body to prevent and treat pediatric diseases (15, 16). Pediatric tuina has been shown to be beneficial for many diseases of infants and children, including the growth problems of preterm infants, painful conditions, musculoskeletal system disorders, psychological problems, neurological conditions, and chronic allergic diseases (such as asthma) (17–24). Several clinical trials have been published in support of the aggressive treatment of AR in children with tuina intervention; however, there is a lack of clear evidence to definitively recommend tuina as a therapeutic option. Therefore, Chinese, English, Korean, and Japanese literature databases were extensively searched for latest published RCTs to systematically evaluate the therapeutic effect of pediatric tuina alone or in combination with other therapies on children with AR with a view to improve clinical practice and further provide evidence for its use.

## Methods

This study has been registered on PROSPERO (CRD 42020220029) and can be accessed at https://www.crd.york.ac.uk/PROSPERO/.

### Inclusion and exclusion criteria

Only randomized controlled trial (RCT) studies were included for meta-analysis. The target study group was children and adolescents aged between 1 and 18 years who had been diagnosed with AR according to established diagnostic criteria. The treatment group received pediatric tuina alone or in combination with other therapies, including acupuncture, Chinese herbal medicine, Chinese patent medicine, and Western medicine. The control group received therapies other than pediatric tuina. Children who received another type of pediatric tuina were excluded. The results included either of the following: effective rate, nasal symptom or sign (nasal congestion, itchy nose, runny nose, sneezing, turbinate swelling, and nasal mucosal swelling) improvement scores, and total nasal symptom scores.

Reviews, meetings abstracts, case reports, comments, and duplicate papers were excluded from the meta-analysis.

### Literature search and study selection

Three English databases (PubMed, Embase, and Cochrane Library), four Chinese databases (Wan Fang Database, China National Knowledge Infrastructure, the Chinese Biomedical Literature Database, and VIP Database for Chinese Technical Periodicals), one Japanese database (cinii), and two Korean databases (Korea citation index, Korean medical database) were searched for relevant literature using the following timeline: from the inception of the coverage of those databases to February 2021. For the English databases, the query strategy comprised three components: clinical condition (allergic rhinitis OR hayfever OR pollinosis), intervention (pediatric tuina OR massage OR manipulation OR manual OR acupressure OR stretching OR touch OR maneuver OR anmo OR chiropractic), and participants (children OR infants OR adolescents OR pediatrics OR toddlers OR preschoolers). For the Chinese, Korean, and Japanese databases, equivalent group terms were queried.

### Data extraction and risk of bias

Two authors (LYT and LRR) independently extracted the data and performed cross-checking. Disagreements, if any, were resolved through discussion. In the event when a consensus could not be reached, the opinion of a third reviewer (WYG) was sought. For each included study, the following clinical features were extracted: 1. study characteristics (title, author, year of publication, country, and sample size); 2. participants (gender, age, and course of disease); 3. interventions; 4. course of treatment; and 5. outcome measurements, which included the nasal symptom or sign score (nasal congestion, runny nose, nasal itching, sneezing, turbinate swelling, and nasal mucosal swelling), total scores for nasal symptoms, and effective rate. To assess the quality of the included studies, two authors (LYT and LRR) independently assessed each study using the Cochrane Collaboration tool for RCTs. Disagreements, if any, between the two reviewers were resolved through discussion and consultation with a third reviewer (WYG), if necessary. In brief, the following seven items of bias were assessed: sequence generation, allocation concealment, blinding of participants and personnel, blinding of outcome assessment, incomplete outcome data, selective reporting, and other deviations. Each domain was rated as being at a low risk, high risk, or unclear risk of bias. Information was retrieved directly from the published articles and supplementary materials and by contacting the study authors when needed.

### Data analysis

The meta-analysis of RCTs with available data was performed using Cochrane Collaboration's Review Manager 5.3. For dichotomous outcomes, the effect size was analyzed *via* relative risk (RR) with 95% confidence intervals (CI). Continuous variable data are expressed as mean difference (MD) with 95% CI. Heterogeneity among the results of the included studies was tested using the chi-square test and combined with I^2^ to quantitatively judge the degree of heterogeneity. *I*^2 ^< 50% and *P* > 0.05 indicated that the test results were not statistically heterogeneous, and the fixed-effects model was used for meta-analysis. By contrast, *I*^2^ > 50% and *P* < 0.1 indicated statistical heterogeneity among the results of the studies, and the random-effects model was used for meta-analysis. *P* < 0.05 indicated statistical significance. If the test results had a medium or high degree of heterogeneity, the source of the heterogeneity was analyzed and solutions such as subgroup analysis or sensitivity analysis were adopted. When the source could not be identified, only descriptive analysis without merging was performed.

## Results

### Study characteristics and risk of bias

**Figure 1:**
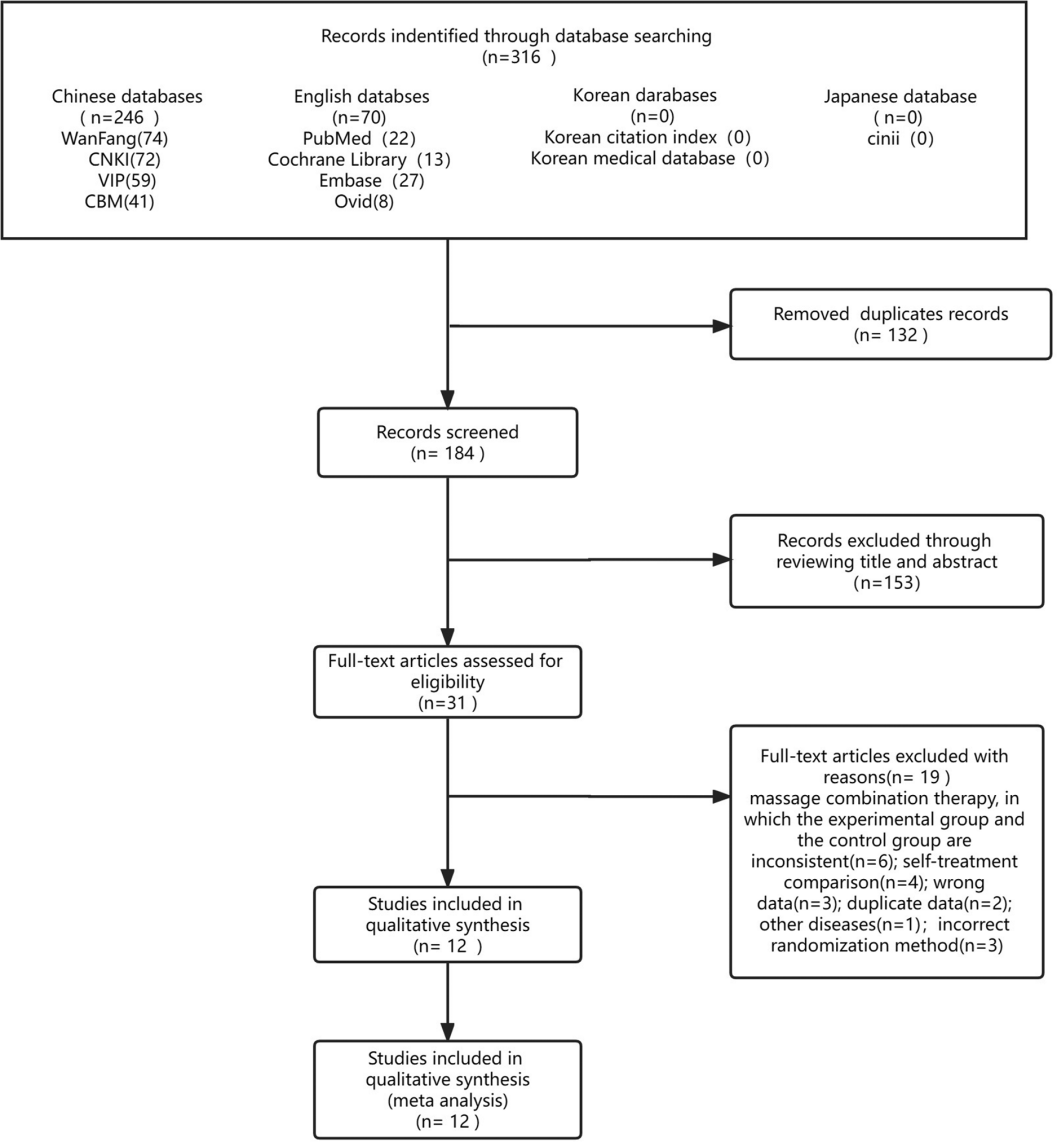
Flowchart for literature inclusion.

A total of 316 potential studies were initially identified, of which 132 duplicate studies were excluded and the remaining 184 articles were further evaluated for specific relevance to the meta-analysis. After further excluding 153 articles deemed irrelevant, 31 articles remained. These 31 articles included 19 studies with incorrect data and inconsistent intervention measures, which were excluded by critically reading the original papers. Finally, a total of 12 studies (25–36), which included 716 children, were included in the meta-analysis. The literature-screening process is shown in Figure 1.

Table 1 summarizes the detailed information of the 12 included studies. These articles were published between 2009 and 2020, and six were published after 2017. Among the 12 included RCTs, 5 (41.67%) studies used pediatric tuina alone to treat AR and 7 (58.33%) used pediatric tuina in combination with herbal medicine (5 articles) and Chinese patent medicine (2 articles) for treatment.

**Table 1:** Characteristics of included studies.

Study	Sample size (M/F)		Age (year)		Course (month)		Interventions		Outcome Measure	Total Duration
	Treatment	Control	Treatment	Control	Treatment	Control	Treatment	Control		
Sun 2018, (25)	30 (16/14)	30 (18/12)	5.1 ± 0.6	4.9 ± 0.7	23.5 ± 11.1	20. ± 9.1	Pediatric tuina	Montelukast	①②③④⑤	3 months
Dai 2020, (26)	28 (15/13)	28 (14/14)	5.7 ± 2.2	5.5 ± 1.9	–	–	Pediatric tuina	Montelukast	①	1 months
Jiang 2017, (27)	30 (18/12)	30 (16/14)	3–5years, 10	3–5years, 12	23.5 ± 11.1	20.9 ± 9.1	Pediatric tuina	Loratadine + Mometasone Furoate Aqueous Nasal Spray	①②④⑤	20 days
			5–8years, 14	5–8years, 11						
			8–10years, 6	8–10years, 7						
Sun 2019, (28)	30 (14/16)	30 (13/17)	3–5years, 10	3-5y, 11	24.6 ± 8.3	22.6 ± 7.0	Pediatric tuina	Montelukast	①②③④⑤	3 months
			5–8years, 9	5-8y, 11						
			8–10years, 11	8-10y, 8						
Fu 2015, (29)	20 (13/7)	20 (12/8)	2.5–6	3-6	5.5-36	6-36	Pediatric tuina	Acupuncture	①	1 months
Xu 2018, (30)	30 (17/13)	30 (20/10)	6.24 ± 1.20	6.3 ± 1.3	25.8 ± 12.3	23.7 ± 13.6	Pediatric tuina + Chinese herbal pieces	Chinese herbal pieces	①③	2 weeks
Chen 2016, (31)	30 (17/13)	30 (20/10)	6.24 ± 1.20	6.3 ± 1.3	25.2 ± 11.7	24.8 ± 13.5	Pediatric tuina + Chinese herbal pieces	Chinese herbal pieces	①	2 weeks
Xu 2009, (32)	40 (20/20)	40 (22/18)	4–10year, 37	4–10years, 37	12–24 m,23	12–24 m,20	Pediatric tuina + Chinese herbal pieces	Chinese herbal pieces	①②	80 days
			>10year, 3	>10year, 3	24–48 m,13	24–48 m,14				
					>48 m,4	>48 m,6				
Shen 2015, (33)	20	20	4.3 ± 1.8	4.9 ± 1.4	23.7 ± 12.9	23.8 ± 14.1	Pediatric tuina + Chinese herbal pieces	Chinese herbal pieces	①②③④	2 weeks
Liu 2014, (34)	60 (34/26)		1.42–7years		4–36		Pediatric tuina + Chinese herbal pieces	Chinese herbal pieces	①	3 months
Fan 2019, (35)	40 (21/19)	40 (20/20)	10.02 ± 1.64	10.8 ± 1.7	36.1 ± 14.	35.2 ± 14.9	Pediatric tuina + Shuangbai Granules		①③	18 days
Mou 2014, (36)	30 (13/17)	30 (15/15)	2–6years, 5	2–6years: 6	–	–	Pediatric tuina + Tongqiao Biyan Tablet	Tongqiao Biyan Tablet	①②	4 weeks
			6–12years, 13	6–12years, 13						
			12–18years, 12	12–18years, 9						

M = Male; F = Female; ER = Effective Rate; NSS = nasal symptom score; TNSS = total nasal symptom scores ① Effective Rate; ② nasal symptom score; ③ total nasal symptom scores; ④ Nasal mucosa swelling score; ⑤ Turbinate swelling score.

**Figure 2:**
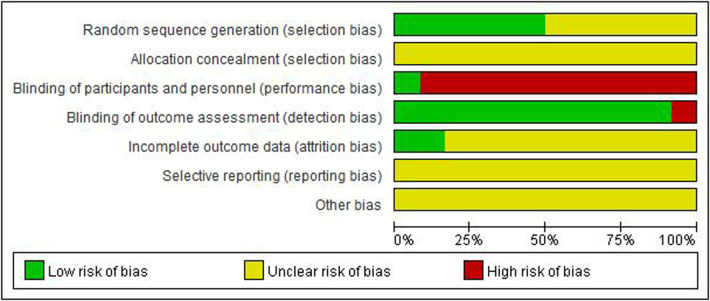
Quality assessment of the trials included in this meta-analysis. Risk of bias graph.

**Figure 3:**
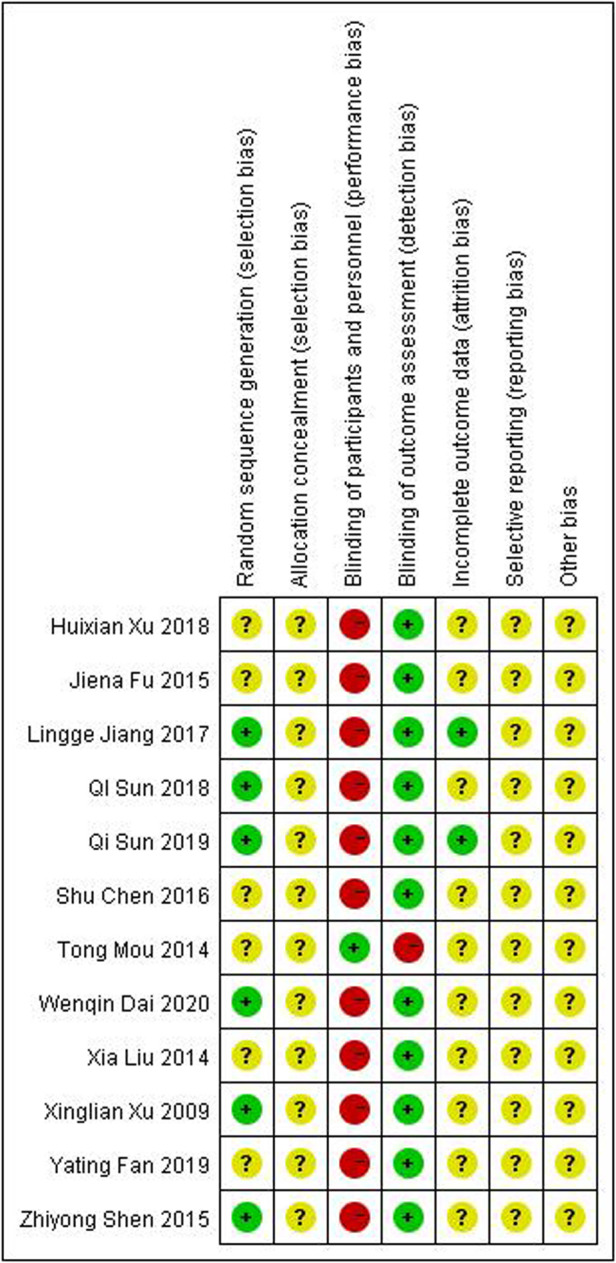
Risk of bias summary.

Figures 2, 3 show an overview of the risk of bias. All studies were randomized: six were randomized using the random number table method and the other six mentioned the use of randomization but did not describe it in detail. Owing to the particularity of pediatric tuina treatment, it is impossible to implement the blinded method for children and tuina experts. Therefore, “blinding of participants and personnel” was deemed “high risk of bias” and “blinding of outcome assessment” was considered “low risk of bias.” Three studies described the dropout data. None of the studies mentioned allocation hiding and selective reporting. Overall, the level of evidence was moderate.

### Outcome evaluation

#### Effective rate

**Figure 4:**
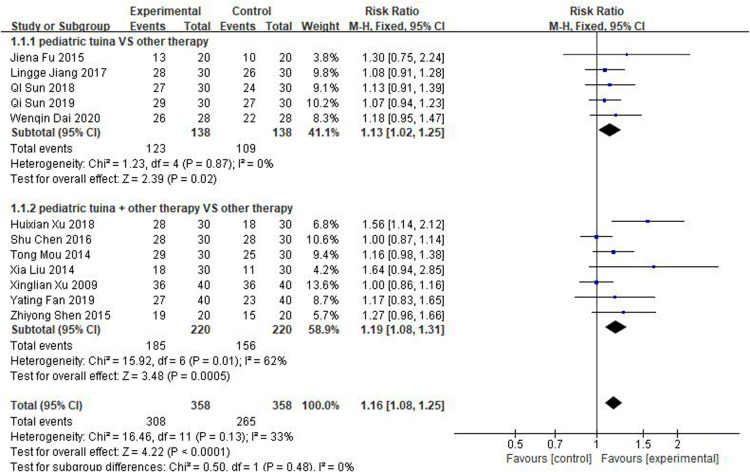
Forest plot of effective rate.

The 12 studies, which included 358 children who received pediatric tuina treatment and 358 children who did not receive tuina in the control group, focused on effective rate as the outcome index. Because of the small heterogeneity of the included studies (*I*^2^ = 33%), a fixed-effect model was used for meta-analysis. The results showed that compared with the control group, pediatric tuina had a higher clinical effectiveness (RR = 1.16, 95% CI:1.08–1.25, *P* < 0.01). Subsequently, a subgroup analysis of five studies using pediatric tuina alone and seven studies using pediatric tuina combined with other treatments was performed. The results revealed that compared with the control group, the effective rate of both pediatric tuina alone (RR = 1.13, 95% CI = 1.02–1.25, *P* < 0.05) or combined with other therapies (RR = 1.19, 95% CI = 1.08–1.31, *P* < 0.05) was superior (Figure 4).

#### Nasal symptom evaluation

**Figure 5:**
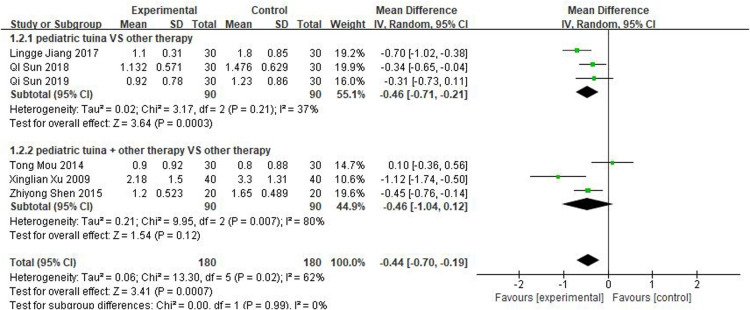
Forest plot for the treatment of nasal congestion symptom.

**Figure 6:**
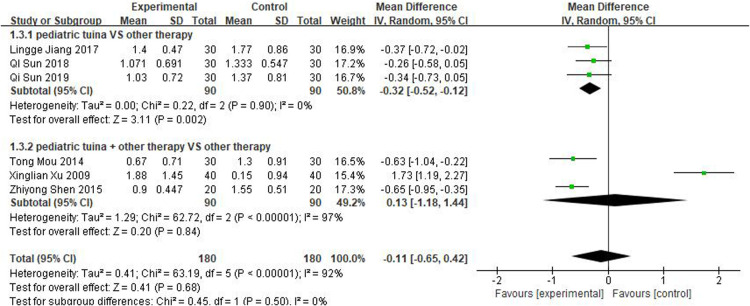
Forest plot for the treatment of nasal itching symptom.

**Figure 7:**
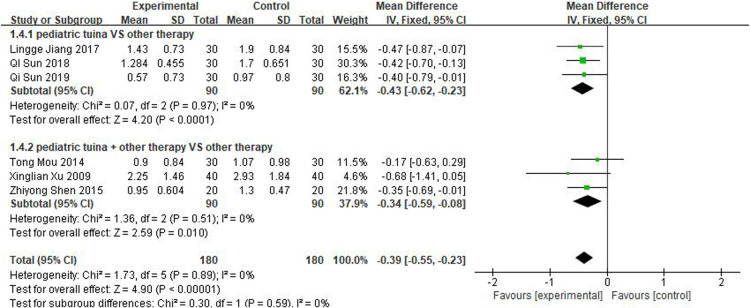
Forest plot for the treatment of runny nose.

**Figure 8:**
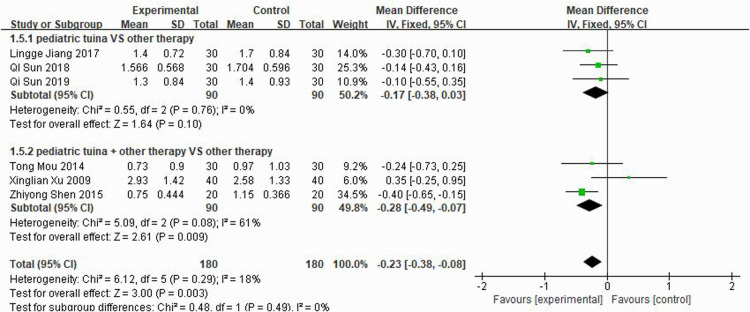
Forest plot for the treatment of sneezing symptom.

**Figure 9:**
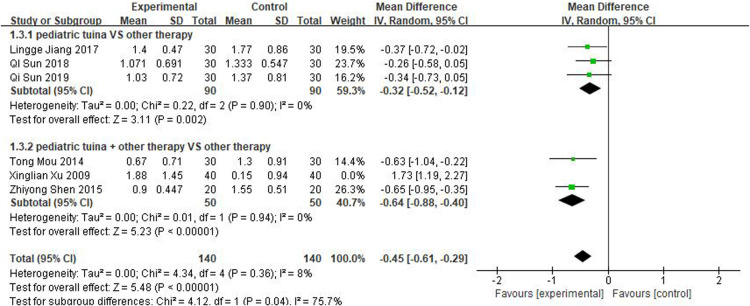
Sensitivity analysis of forest plot for the treatment of nasal itching symptom.

Nasal symptoms included nasal congestion, itchy nose, sneezing, and runny nose. Six articles evaluated the four symptoms separately, three of which were with pediatric tuina alone and three with pediatric tuina combined with other treatments. A total of 180 children who received pediatric tuina and 180 controls were included in the meta-analysis of the four symptoms of nasal congestion (Figure 5), nasal itching (Figure 6), runny nose (Figure 7), and sneezing (Figure 8). The results revealed that both pediatric tuina alone or combined with other therapies significantly improved nasal congestion (MD = −0.44, 95% CI = −0.70 to −0.19, *P* < 0.01), runny nose (MD = −0.39, 95% CI = −0.55 to −0.23, *P* < 0.01), and sneezing (MD = −0.23, 95% CI = −0.38 to −0.08, *P* < 0.01) but not nasal itching (*P* = 0.68). The results of the aggregated data showed that the heterogeneity of pediatric tuina treatment for nasal congestion and nasal itching was relatively high (*I*^2^ = 62% and 92%, respectively), which may be related to the inconsistency of the nasal symptom score scale used by Xu et al. (32). Further sensitivity analysis was performed by eliminating this article with respect to nasal itching, and the results revealed that the heterogeneity was only 8% (Figure 9; MD = −0.45, 95% CI = −0.61 to −0.29, *P* < 0.01).

#### Total nasal symptom evaluation

**Figure 10:**
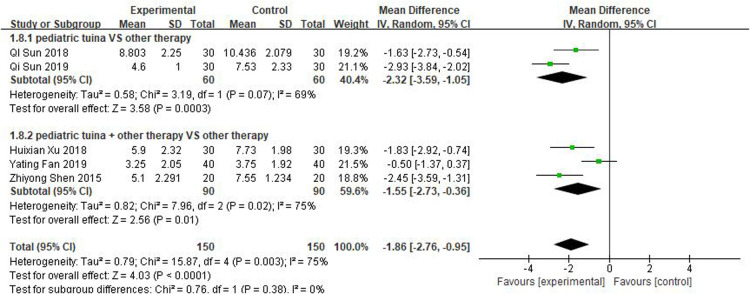
Forest plot of the total scores for the treatment of nasal symptoms.

Scores for total nasal symptoms were reported in 5 studies involving 150 children treated with pediatric tuina and 150 controls. The meta-analysis revealed that pediatric tuina was superior to other therapies in improving nasal symptoms (MD = −1.86, 95% CI = −2.76 to −0.95, *P* < 0.01). The heterogeneity of the included studies was relatively high (*I*^2^ = 75%), so a random-effects model was used. The results of the subgroup analysis showed that compared with the control group, pediatric tuina alone (MD = −2.32, 95% CI = −3.59 to −1.05, *P* < 0.01) or combined with other therapies (MD = −1.55, 95% CI = −2.73 to −0.36, *P* < 0.01) exhibited better overall symptom score and improvement (Figure 10).

#### Nasal sign evaluation

**Figure 11:**
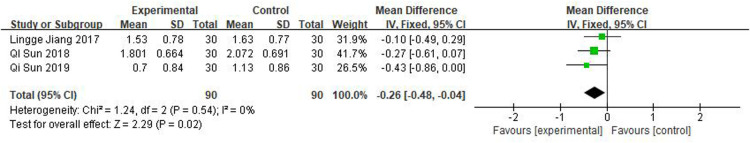
Forest plot showing the signs of turbinate swelling.

**Figure 12:**
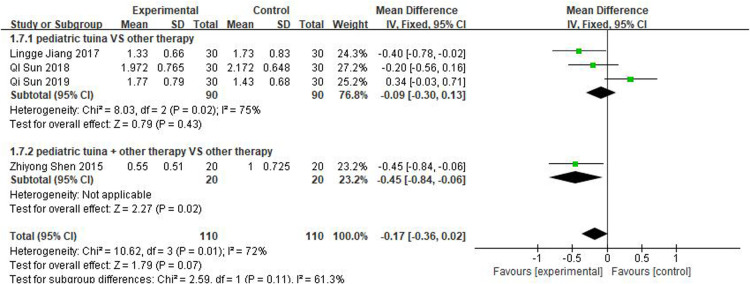
Forest plot for signs of nasal mucosal swelling.

Three studies rated turbinate swelling and four rated nasal mucosal swelling. The results of these studies revealed that pediatric tuina was superior to the control group in improving turbinate swelling (Figure 11; MD = −0.26, 95% CI = −0.48 to −0.04, *P* < 0.01), with low heterogeneity *I*^2 ^= 0%. However, compared with the control group, pediatric tuina exhibited no significant advantage in improving nasal mucosal swelling (Figure 12; MD = −0.17, 95% CI = −0.36 to 0.02, *P* = 0.07).

#### Publication bias

**Figure 13:**
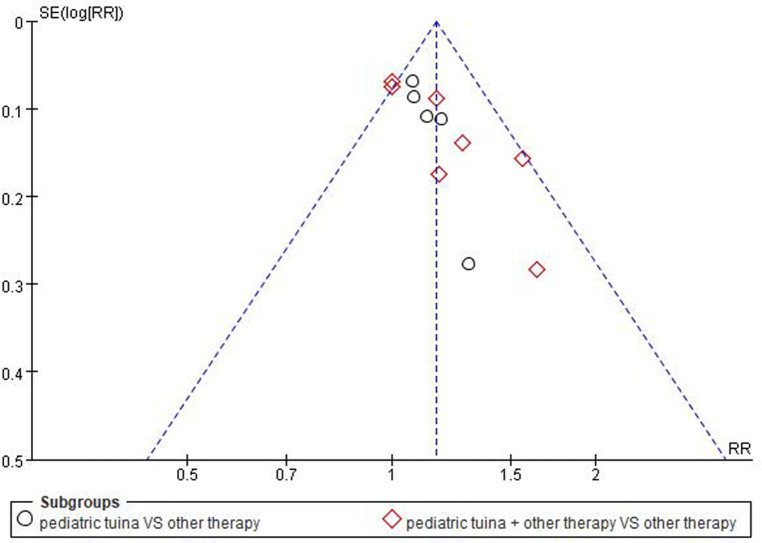
The funnel plot of publication bias.

As the number of included RCTs was >10, a funnel plot was used to analyze the risk of publication bias. The result showed that the included studies were concentrated in the middle and top of the graph and the left–right distribution was basically symmetric, suggesting that the included studies had low publication bias (Figure 13).

## Discussion

The present meta-analysis included 12 published RCTs, 50% of which were published in the last 5 years. A previously published systematic review (37) provided some suggestive evidence that pediatric tuina may be beneficial in the treatment of AR in children. However, this systematic review primarily included Chinese databases and two English databases, excluding foreign language databases. Moreover, nearly 5 years have passed since its publication, and some follow-up latest clinical trials have not yet been analyzed. Compared with the previous meta-analyses, eight recent RCTs were included in the present analysis after an extensive search of Chinese, English, Korean, and Japanese databases. Pediatric tuina was considered the standard or adjunctive treatment for AR in children in all these trials. All 12 articles included in the study used the total effective rate as the outcome indicator, and the results revealed that compared with other therapies, pediatric tuina was more effective in treating children with AR. For the unsatisfactory effect of single medication, Allergic Rhinitis and its Impact on Asthma guidelines recommend the use of a combination regimen to treat children with AR (9). Current studies revealed that pediatric tuina combined with Western medicine, acupuncture, and traditional Chinese medicine was superior to a monotherapy in the treatment of pediatric systemic diseases (38–40). Consistent with the results of these studies, the present study confirmed that pediatric tuina combined with traditional Chinese herbal medicine was more effective in AR treatment.

The score of nasal symptoms and signs represents the severity of AR and is the most commonly used outcome evaluation indicator for AR (41). The subgroup analyses in the current study provided some confirmation that pediatric tuina as a physiotherapy is superior to drugs or acupuncture in the relief of nasal symptoms scores such as nasal congestion, nasal itching, sneezing, runny nose, and turbinate swelling in pediatric patients with AR. Pediatric tuina for children with AR is based on the principle of combining local and remote acupoint selection and characterized by the local manipulation of the nose. The literature included in this study all implemented pediatric tuina on common nasal acupoints (such as Yingxiang, Bitong, Yintang, and others) to improve nasal redness, hot flashes, and local qi and blood circulation. Lei et al. also confirmed that the pediatric tuina of Bitong and Yingxiang significantly improved the nasal symptoms of children with AR (42). The included literature applied distal acupoint selection to treat children with AR, particularly the special acupoints below the elbows and knees of the limbs, including the lung meridian and spleen meridian (29, 30). By pushing and rubbing these acupoints, nasal allergy symptoms and immunity can be improved (43). The analysis showed that there is a high degree of heterogeneity in the improvement of nasal symptoms and signs with tuina. This may be attributed to inconsistencies in the rating scales used. In addition, different manipulation schemes (such as the selected acupoints as well as the time and frequency of manipulation) are another source of high heterogeneity.

Among the 12 articles included in this study, only 3 articles reported adverse reactions. The analysis showed that there were no significant adverse reactions during the treatment of AR with pediatric tuina. Therefore, a meta-analysis of adverse reactions was not performed. Clinically, the most common adverse reaction of pediatric tuina is skin damage, which is mostly caused by improper manipulation. Skin damage can be avoided by adjusting different manipulation strengths according to the child's body constitution.

Despite the finding that pediatric tuina has significant clinical benefits in AR, the underlying mechanisms of its therapeutic action remain largely unexplored. Pediatric tuina and acupuncture therapy are guided by the basic principles of TCM using the theory of meridians and acupoints as the core. The clinical location of acupoints in the treatment of AR in children is roughly the same. The pediatric tuina treatment of AR employs fingers instead of acupuncture needles to manipulate meridian acupoints and trigger meridian conduction. Thus, pediatric tuna may have a similar mechanism of action to acupuncture in the treatment of AR, i.e., the stimulation of acupuncture points could activate the autonomic nervous system (44), trigger neural reflexes in the immune system, and decrease inflammatory cytokine and IgE levels (45, 46).

The efficacy of pediatric tuina has a certain correlation with age and intervention courses. The publication age of papers included in the present analysis ranged from 1 to 18 years. According to the Chinese Medical Encyclopedia, pediatric tuina is suitable for children aged <6 years, particularly for infants aged <3 years. However, optimal efficacy is based on the clinical experience of pediatric tuina experts. At present, there is still a lack of studies on the efficacy of pediatric tuina in different age groups, and additional clinical evaluations are needed in the future.

The present meta-analysis study has some limitations. First, there were multiple heterogeneities among the included trials regarding the type of AR, basic treatment (e.g., pharmacotherapy, traditional Chinese medicine, and acupuncture), scoring of nasal symptoms, and variations in pediatric tuina procedures. Additional studies are needed to fully assess how these factors play a role in heterogeneity. Second, because the interventions of pediatric tuina were completely different from those of the control group, a relatively high risk of bias existed owing to the lack of blinding.

## Conclusion

The present study revealed that pediatric tuina is a safe and effective treatment for AR in children as it can effectively relieve the nasal symptoms of children. Thus, pediatric tuina is worth promoting in clinical practice. Considering the small sample size and lack of follow-up data of the included studies, more multicentric RCTs with a large sample and sufficient follow-up duration are needed to validate these findings.

## Data Availability

The original contributions presented in the study are included in the article/Supplementary Material, further inquiries can be directed to the corresponding author/s.
